# Senolytic Treatment Improve Small Intestine Regeneration in Aging

**DOI:** 10.14336/AD.2023.0920

**Published:** 2024-08-01

**Authors:** Qing-Tian Luo, Yuan-Chun Ye, Wei-Ming Guo, Qing Zhu, Sa-Shuang Wang, Nan Li, Lei Wang, Chun-Sheng Cheng, Gang Fan

**Affiliations:** ^1^ Department of Gastroenterology, Huazhong University of Science and Technology Union Shenzhen Hospital, the 6^th^ affiliated Hospital of Shenzhen University Medical School, Shenzhen, Guangdong, China.; ^2^ Department of Gastroenterology, Quanzhou First Hospital Affiliated to Fujian Medical University, Quanzhou, Fujian, China.; ^3^ Sports Medicine Center, Huazhong University of Science and Technology Union Shenzhen Hospital, the 6^th^ affiliated Hospital of Shenzhen University Medical School, Shenzhen, Guangdong, China.; ^4^ Pain Management Department of The Second Affiliated Hospital, School of Medicine, The Chinese University of Hong Kong, & Longgang District People's Hospital of Shenzhen, Shenzhen, Guangdong, China.; ^5^ Department of Pain Medicine and Shenzhen Municipal Key Laboratory for Pain Medicine, Huazhong University of Science and Technology Union Shenzhen Hospital, the 6^th^ affiliated Hospital of Shenzhen University Medical School, Shenzhen, Guangdong, China.; ^6^ Department of Orthopedics, Shanghai Sixth People's Hospital, Shanghai Jiao Tong University, Shanghai, China.; ^7^ Centre for Musculoskeletal Surgery, Charité - Universitätsmedizin Berlin, Corporate Member of Freie Universität Berlin, Humboldt-Universität zu Berlin, and Berlin Institute of Health, Berlin, Germany.; ^8^ Urology Department, Huazhong University of Science and Technology Union Shenzhen Hospital, the 6^th^ affiliated Hospital of Shenzhen University Medical School, Shenzhen, Guangdong, China.

**Keywords:** senolytic, aging, small intestine, villus, crypt, Ki67

## Abstract

Aging induces a series of alterations, specifically a decline in the stature and number of villi and crypts in the small intestine, thus compromising the absorbent capability of the villi. This investigation employed a senolytic combination of dasatinib and quercetin (D+Q) to examine its impact on the intestinal tract of elderly mice. Our findings demonstrate that D+Q treatment leads to a decrease in the expression of p21, p16, and Ki67, while concurrently triggering removal of apoptotic cells within the villi. Additionally, D+Q treatment exhibits the ability to promote growth in both the height and quantity of villi and crypts, along with stimulating nitric oxide (NO) production in aged mice. The study presented a model to assess strategies to alleviate age-related senescence in the intestinal tract of elderly mice. Importantly, D+Q showcases promising potential in enhancing intestinal functionality within the aging.

Aging is a complex biological process and associated with a progressive decline in functions of most organs [1]. Taking gastrointestinal (GI) tract as an example, aging can lead to intestinal barrier dysfunction [2], enteric neuromuscular systems dysfunction [3], and even degeneration of intestinal villi [4]. Intestinal villi are composed of a layer of intestinal epithelia cells (IECs) and the lamina propria, and their main function is to absorb nutrients, and the absorption activity is affected by the size and the density of villi [5]. The epithelial layer is renewed every 4-5 days by intestinal stem cells (ISCs) in the intestinal crypt according to a symmetrical division and neutral shift pattern, which generate transient amplifying (TA) progenitor cells that later differentiate into absorptive or secretory cells [6]. The density of crypts and villi are controlled by ISC fission [6]. It has been reported that aging leads to a decrease in the size and density of villi and crypts in mice, which may be related to the inhibition of ISC proliferation during aging [7]. Therefore, nutrient malabsorption is very common among the elderly, and often causes fatigue, edema, anemia and other illnesses [8]. Thus, preventing the aging of intestinal villi is of great significance for improving nutrient absorption disorders in the elderly.

Senescent cells (SCs) amass in numerous tissues during the aging process and in locations associated with the development of various chronic illnesses [9]. Convincing experimental and clinical data support the notion that targeting cellular senescence could potentially impede the aging process and ameliorate age-related ailments [10]. Several senolytic agents have been discovered, which efficiently target distinct prosurvival pathways, including BCL-2/BCL-xL, p53/p21, and PI3K/AKT, as well as antiapoptotic pathways involving serpins. Pharmacological inhibitors of these proteins induce cell death in both murine and human senescent cells. Additionally, these agents demonstrate senolytic activity *in vivo* when specifically directed at these pathways [11, 12]. D+Q, a combination treatment of dasatinib (D), a tyrosine kinase inhibitor, with quercetin (Q) has been best studied of senolytics in many diseases or disorders, such as to preserve cell viability, phenotype, and matrix content in age-dependent intervertebral disc degeneration [13] and to depleted callus SCs and accelerated fracture healing in fractures in old mice [14]. D+Q present highly clinical translational potential since both drugs have approved for use in human and have been demonstrated relative safety with oral administration [15]. Clinical trials with D+Q on aged-related disease have been extensively examined in recent years, its anti-aging protective effect has been observed and appeared promising in patients with idiopathic diabetic kidney disease [16], pulmonary fibrosis [17], and Alzheimer’s disease [18]. The role of D+Q in GI aging has been rarely studied. A recent study has showed D+Q treatment reduced senescence markers levels and inflammation in ileum, caecum and colon while altering specific microbiota signatures in aged mice [19]. However, the effect of D+Q on epithelial structure and growth of the small intestinal epithelium in aged mice remains unknown. Since its safety profile has already been proven [15], in this study, we investigated the effect of the senolytic cocktail dasatinib plus quercetin on the epithelium of the aging small intestine.

## MATERIALS AND METHODS

### Mouse models

Young (3-month-old)-, Old (vehicle-treated) (16-month-old)-, D+Q treated old (16-month-old) male C57BL/6 mice were used. Mice were purchased from Shenzhen TopBiotech Co., Ltd and maintained at the breeding facility of the Animal Center of Huazhong University of Science and Technology Union Shenzhen Hospital in individually ventilated cages under standardized conditions that included a 12-hour dark-light cycle and free access to standard chow, and water *ad libitum*. Animal experiments were carried out in accordance with recommendations in the National Research Council Guide for Care and Use of Laboratory Animals and in comply with relevant ethical regulation for animal testing and research, with the protocols approved by the Institution Animal Care and Use committee of Huazhong University of Science and Technology Union Shenzhen Hospital. There are no ethical concerns.

### Drug administration

Mice were administered a senolytic cocktail containing 5 mg/kg dasatinib (Selleck Chemicals, S1021) and 50 mg/kg quercetin (Sigma-Aldrich, Q4951) as previously described [20]. Briefly, Dasatinib and quercetin were dissolved in 10% polyethylene glycol 400 (PEG 400; Sigma-Aldrich, #25322-68-3). Mice were gavaged bi-weekly for 4 months with D+Q or vehicle (10% PEG 400).

### Histological analysis and immunohistochemistry (IHC)

Small intestines were harvested immediately after killing and washed with PBS. The jejunum was opened longitudinally and coiled with the mucosal layer inward using a wooden stick like Swiss Rolls and then fixed in 4% paraformaldehyde. Tissues were dehydrated, cleared and embedded in paraffin, cut into serial 5-μm, and the sections were stained with hematoxylin for 2 min and eosin for 5 min at room temperature. IHC was performed as described in our previous study [21]. Formalin-fixed and paraffin-embedded tissue blocks were cut into 5-μm-thick sections and mounted on glass slides. Slides were heated in an oven at 70˚C for 90 min and deparaffined in xylene twice for 10 min each at room temperature, rehydrated in a descending ethanol series (100, 95 and 85% ethanol for 5 min each at room temperature) and incubated in 3% H_2_O_2_ for 8 min at room temperature to block endogenous peroxidase. Antigen retrieval was performed by heating in a microwave at 100˚C in sodium citrate buffer (3 mM, pH 6.0) for 15 min. Slides were blocked with 5% bovine serum albumin (Beijing Solarbio Science & Technology Co., Ltd.) for 1 h at room temperature to block non-specific antibody binding and incubated overnight at 4˚C with primary antibody against Ki67 (Sevicebio, GB111141, 1:100). In this step, negative controls are incubated with antibody dilution (cat. no. G2025-100ML; Servicebio) instead of primary antibody. Negative controls were employed to validate antibody specificity and distinguish genuine target staining from background. Following the primary antibody incubation, the sections were washed three times with PBS and incubated with horseradish peroxidase (HRP) secondary anti-rabbit antibodies (ready to use; cat. no. PV-6000; OriGene Technologies, Inc.) at 37˚C for 30 min. The sections were stained with 3,3’-diaminobenzidine at room temperature; the duration of staining was based on the staining observed under a light microscope with a magnification of x100, and the reaction was terminated when the staining was yellowish-brown. The sections were then counterstained with hematoxylin for 1 min at room temperature. 10-15 fields were randomly selected from each section at high magnification of x100, photographed Nikon DS-U3-NIS-Elements microscope and analyzed double blindly by two researchers.

### TUNEL assay

The presence of DNA strand breaks was evaluated by TUNEL assay using TMR (red) Tunel Cell Apoptosis Detection Kit (Servicebio), according to the manufacturer’s protocol described subsequently. Formalin-fixed and paraffin-embedded tissue blocks were cut into 5-μm-thick sections and mounted on glass slides. Slides were deparaffined in xylene twice for 15 min each at room temperature, rehydrated in a descending ethanol series (100, 95, 90, 85 and 70% ethanol for 5 min each at room temperature). Antigen retrieval was performed by adding proteinase K solution to cover objectives and incubating at 37˚C for 25 min. The cell permeability was done using 0.1% Triton X-100 at room temperature for 20 minutes and then incubating with the TUNEL reaction mixture (TDT enzyme: dUTP: buffer = 1:5:50) in a humidified chamber and dark room at room temperature for 2 hours. After washing in PBS, the slides were counterstained with 1 mg/ml 4, 6- diamidino2-phenylindole (DAPI) (Servicebio) solution at room temperature for 10 min in dark place. After washed with PBS three times, the slides were mounted with anti-fade mounting medium. Positive apoptosis cells are red. 10-15 fields were randomly selected from each section at high magnification of x100, photographed using Nikon Eclipse C1 microscope, and analyzed double blindly by two researchers.

### Epithelial cell and crypt isolation

Epithelial cell and crypt isolation was performed as previously described [7]. In brief, the jejunum was removed immediately, and the stool was flushed out with ice-cold PBS. The tissues were dissected and opened longitudinally and cut into 1-cm pieces, which were placed in PBS with 2 mmol/EDTA for 30 min at 4°C and then in PBS with 54.9 mmol/L d-sorbitol and 43.4 mmol/L sucrose. The pieces were then vortexed for 1-2 min, filtered by a 70 μm sterile cell strainer. The epithelial cells and crypts were enriched by centrifugation at 150 g for 10 min at 4°C.

### Western blot analysis

Western blot analysis was performed as described in previous study [21] .The isolated jejunal epithelial cells were homogenized in ice-cold RIPA lysis buffer (Beijing Solarbio Science & Technology Co., Ltd.) containing protease and phosphatase inhibitors and 1 mM PMSF. Protein concentrations of the lysates were estimated following centrifugation at 12,000 x g for 15 min at 4˚C and quantified using a bicinchoninic acid protein assay kit (Beijing Solarbio Science & Technology Co., Ltd.). 15 μg of proteins were loaded for each lane and separated by 12% SDS-PAGE gel, and then transferred onto 0.2 μm polyvinylidene difluoride membranes (EMD Millipore). After the transfer, the blots were blocked with 3% BSA for 1 h at room temperature and then incubated with the primary antibodies at 4 °C overnight. The following primary antibodies were used: rabbit anti-p16 (Beyotime, AF1069, 1:500) and mouse anti-p21 (Beyotime, AP021, 1:1,000). For loading control, the blots were probed with mouse anti-GAPDH (Affinity Biosciences, AT0004, 1:1000) or rabbit anti-β-Actin antibodies (Servicebio GB11001, 1:1000). These blots were further incubated with corresponding HRP-conjugated goat anti-rabbit (Abcam, ab205718, 1:5000) or goat anti-mouse secondary antibodies (Abcam, ab205719, 1:5000) at 4˚C for 4 h. Protein bands were visualized using an ECL reagent (Thermo Fisher Scientific, Inc.) on a FluorChem E system (ProteinSimple, Inc). Specific bands were evaluated by apparent molecular sizes. Relative expressions were used to analyze the results. The grey values of all the specific bands were semi-quantified using NIH Image J software, and the grey values of each target protein band were compared with the grey values of the corresponding internal reference proteins to derive a normalized grey value, and the mean of the normalized grey values of the bands in the young group was set to 1. The normalized grey value of each band was compared with the mean of normalized gray values in young group to determine the relative expression.

### Villi and crypt quantitation

Villi and crypt quantitation were measured as previously described [7]. The number of villi per view (10 × 10 magnification) were counted double blindly. The height of villi and crypts were measured from top to the bottom using Nikon DS-U3-NIS-Elements microscope. Each group has at least three mice, and the results represent an average of 5 sections per mice.

### RNA isolation, RNA sequencing and function enriched analysis

RNA isolation, RNA sequencing and function enriched analysis were performed as described previously [22]. The isolated jejunal epithelial cells from young, vehicle- and D+Q-treated mice were used to isolate RNA with Trizol (Invitrogen, CA, USA) according to the manufacturer’s protocol. RNA concentration was determined by utilizing a Nanodrop 2000 (Thermo Fisher) and RNA integrity was quantified using an Agilent Bioanalyzer 2100 (Agilent Technologies, Santa Clara, CA). High-quality RNA (RIN >8.0) of 500 nanograms was then sent to Novogene (Beijing, China) for sequencing. RNAseq analyses were carried out using Partek® Flow® software, v10.0 (St. Louis, MO). The default QA/QC tool was employed for pre-alignment quality control. The splice aware program STAR (v2.7.8a) was used for aligning sequencing reads to the mouse genome (GRCm39). Gene counts were quantified using Partek E/M against transcriptome release 103 with a minimum expression cutoff of 10 counts to filter out low expression genes. Differential gene expression analysis between the D+Q and old mice groups was performed using the R program DESeq2 package, considering a *P*-value <0.05 and an absolute log-fold change (logFC) >0.5 as the thresholds. To explore the potential biological functions associated with the different groups, Gene Set Enrichment Analysis (GSEA) was conducted using the clusterProfiler package in R, using a significance threshold of *P*-value <0.05.

### Nitric oxide assay

Nitric oxide assay was performed as described in previous study [23]. Mice were deeply anesthetized with isoflurane and perfused through the ascending aorta with 20-25 ml PBS. Following perfusion, jejunum was harvested immediately after killing and washed with ice-cold PBS and cut into 1.5-cm pieces. Tissues were then embedded in OCT medium (Tissue-Tek) and cryosectioned to produce 20 μm-thick sections, which were mounted onto charged slides. Slides were heated in an oven at 65˚C for 90 min and washed with PBS for three times at room temperature. Then, the slides were incubated with 5 μM DAF-FM DA (Beyotime Biotechnology) at 37˚C for 30 min and washed with PBS for three times at room temperature. The 3-5 fields were randomly selected from each section at high magnification of x100, photographed using ThermoFisher Invitrogen EVOS M5000 system, and analyzed double blindly by two researchers.

### Statistical analysis

All data are reported as the mean ± SEM as indicated in the figure legends. In most cases, each data point corresponds to an individual animal. All experimental data shown has been reliably reproduced by multiple lab members. All biochemical data were statistically analyzed with GraphPad Prism 9.0 software. Whether the data were normally distributed was tested using the Shapiro-Wilk normality test. A one-way ANOVA followed by a Tukey’s post hoc test was used to determine the statistical differences. If the data were not normally distributed, comparisons were performed by non-parametric test. *P*-values < 0.05 were considered statistically significant.

### Senolytic alleviate senescence of small intestine

**Figure 1.**
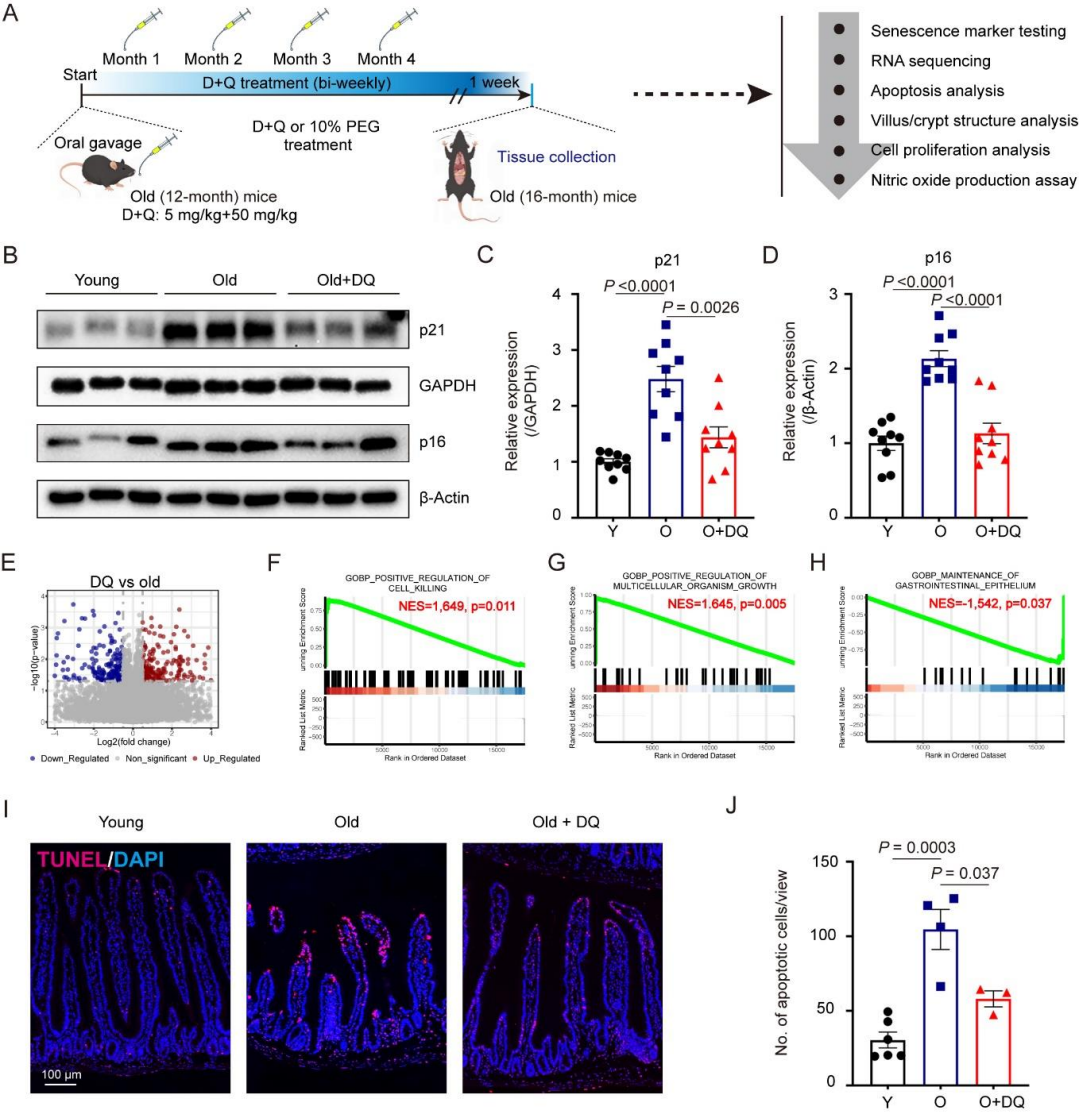
**D+Q rescue senescence in small, aged intestine**. (**A**) Experimental design of senolytic administration and experimental arrangement is shown. (D, dasatinb; Q, quercetin; PEG, polyethyene glycol). (**B**) Western blot showing an up-regulation of p21 and p16 expression in the isolated jejunal epithelial cells in old mice, which were rescued by D+Q treatment. (**C-D**) Semi-quantification of expression levels of p21 (C) and p16 (D) present in part (B) was performed using ImageJ software. GAPDH or β-Actin were detected as a loading control and for normalization. *n*=3 mice in each group. The experiment was repeated three times. (**E-H**) Volcano plot of RNA-seq data of 472 genes from vehicle-, and D+Q treatment mice (n = 3 samples for each group). F-H, shown are gene set enrichment analyses of positive regulation of cell killing (F), positive regulation of multicelluar organism growth (G) and maintenance of gastrointestinal epitheliun (H) in D+Q compared with vehicle group. (**I**) TUNEL assay showing the apoptotic cells in jejunal from a 3-month-old mouse vs. vehicle- vs. D+Q treated 16-month-old mouse. Red, TUNEL; blue, DAPI. Scale bar, 100-μm. (**J**) Quantification data in part (I). *n* =6 in young mice group, *n* =4 in vehicle treated old mice group and *n* =3 in D+Q treated old mice, respectively. Data are expressed as mean ± SEM. *P* values were determined using a one-way ANOVA followed by a Tukey’s post hoc test (Fig. 1C-D, J). Y, young; O, Old; DQ, dasatinib and quercetin.

To investigate the impact of D+Q on small intestine in aging, we collected jejunum from 3-month-old mice and 16-month-old mice treated with/without D+Q (Fig. 1A). Dasatinib (D) is an inhibitor of multiple tyrosine kinases and is known to interfere with EFNB-dependent supprepression of apoptosis [24, 25]. Quercetin (Q), a natural flavonol, inhibits PI3K, other kinases, and serpines [26, 27]. The co-administration of compounds D and Q triggers apoptosis in specific types of senescent cells and shows potential in mitigating senescence-related phenotypes in mice [28]. As p21 (encoded by *Cdkn1a*) and p16 (encoded by *Cdkn2a*) were identified as biomarkers of cellular senescence [29]. Upon cellular entry into senescence, phenomena driven by the activation of the p53/p21 and p16/pRB signaling pathways manifest, culminating in the establishment of cell cycle arrest, increased levels of p16 and p21 are often used to identify cells with senescence-associated phenotypes [30, 31]. Therefore, in this study, we firstly investigated the expression of p21 and p16 in small intestinal epithelium treated with either vehicle- or D+Q. Consistent with the idea that the aged small intestinal epithelium samples showed increased expression of p21 and p16 proteins, which were suppressed by D+Q treatment in 16-month-old mice (Fig. 1B-D). The result is consistent with the previous study in aging mice that D+Q-treated aged mice exhibit a pronounced reduction in senescent cell presenting by diminished levels of p16 and p21 expression within both the small and large intestine, when compared to the control mice [19]. Given the absence of untoward consequences in mice resulting from the protracted administration of senolytic drugs D+Q, these findings underscore the advantageous safety profile of D+Q therapy [13]. Henceforth, our investigation primarily sought to assess the consequential influence of D+Q treatment specifically on the small intestine.

**Figure 2.**
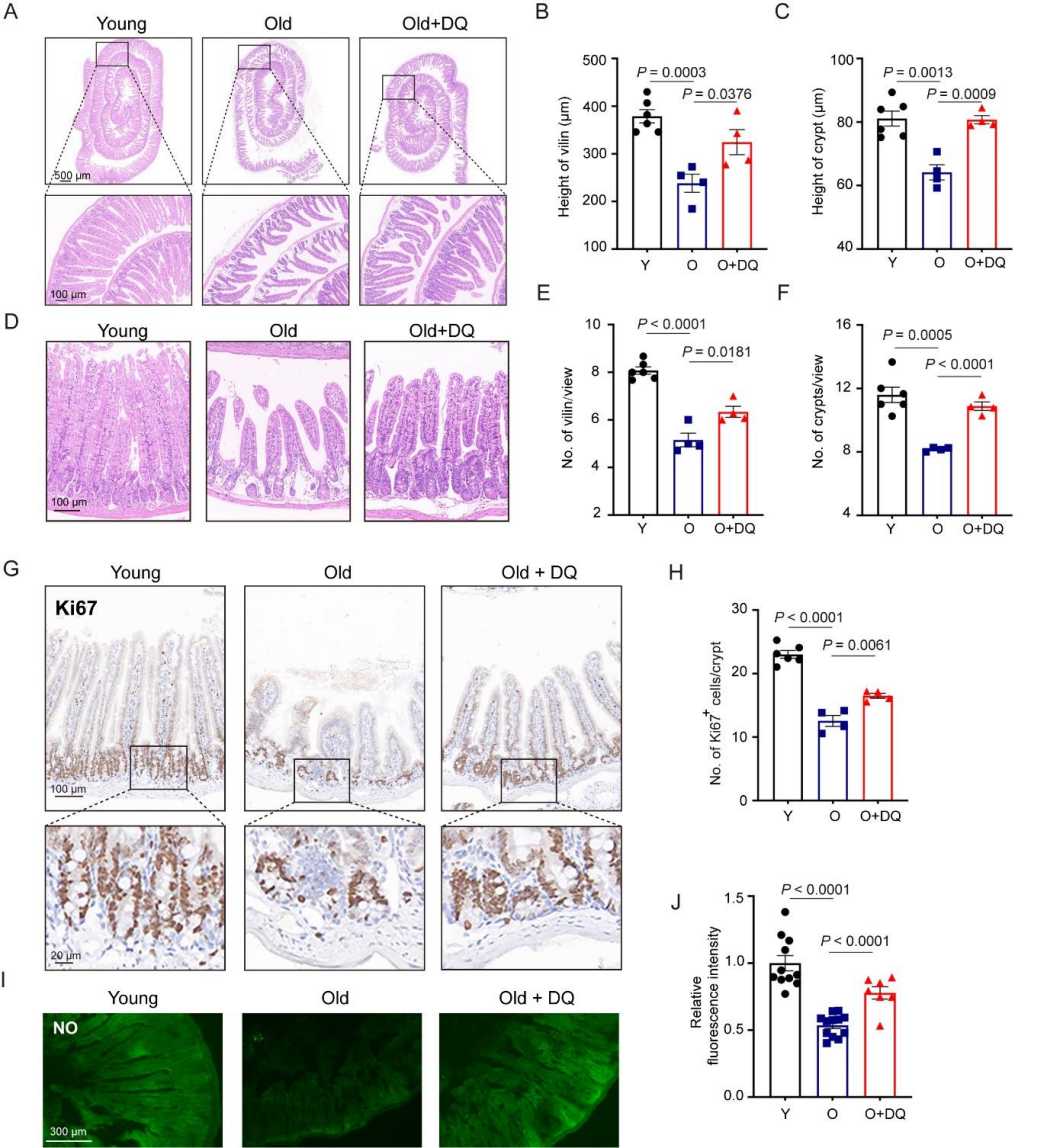
**D+Q promotes villus/crypt structure and nitric oxide production in old, small intestine**. (**A-D**) Old mice showed deterioration in villus and crypt structures, which were rescued by D+Q treatment. (**A**) Old mice showed decreases in height of villi and crypts, which were rescued by D+Q treatment. Scale bars, 500-μm (upper) and 100-μm (lower). (**B-C**) Quantitation data in part (A). (**D**) Old mice showed decreases in number of villi and crypts, which were rescued by D+Q treatment. Scale bar, 100-μm. (E and F) Quantitation data in part (D). *n* =6 in young mice group, *n* =4 in vehicle treated old mice group and D+Q treated old mice, respectively. (**G**) Old mice showed a decrease in the number of proliferating cells in crypts, which were rescued by D+Q treatment. Scale bars, 100-μm (upper) and 20-μm (lower). (**H**) Quantitation data of number of Ki67 positive cells per crypt in part (G). *n* =6 in young mice group, *n* =4 in vehicle treated old mice group and D+Q treated old mice, respectively. (**I**) Nitric oxide assay showed a decrease in NO content in small intestine in old mice, which was rescued by D+Q treatment. Scale bar, 300-μm. (**J**) Quantitation data of relative fluorescence intensity in part (I). *n* =6 in young mice group, *n* =4 in vehicle treated old mice group and D+Q treated old mice, respectively. Data are expressed as mean ± SEM. *P* values were determined using a one-way ANOVA followed by a Tukey’s post hoc test (Fig. 2B-C, E-F, H, J). NO, Nitric oxide; Y, young; O, Old; DQ, dasatinib and quercetin.

In order to further elucidate the effects of D+Q on the old small intestine, we conducted an analysis of RNA sequencing data obtained from the intestinal epithelial cells of 16-month-old mice. These mice were subjected to treatment with either a control vehicle or D+Q compound. We identified a total of 472 genes displaying differential expression levels between the intestinal epithelial cells of 16-month-old mice subjected to either the senolytic-, or vehicle-treated conditions (Fig. 1E). Utilizing Gene Set Enrichment Analysis (GSEA), we additionally discovered a plethora of signaling molecules and genes associated with the response to D+Q treatment, including positive regulation of cell killing, positive regulation of multicelluar organism growth and maintenance of gastrointestinal epitheliun in D+Q compared with vehicle group (Fig. 1F-H). Gut epithelial cells are characterized by rapid, constant cell renewal [32]. The disposal of aging epithelial cells around the villus tips of the small intestine occurs regularly and it has been regarded as a consequence of well-controlled cell death, designated as apoptosis [33]. To address whether D+Q treatment contribute to the apoptosis in old small intestinal, we analysed the expression of TUNEL in small intestinal from 16-month-old mice treated with D+Q or vehicle. The findings of the TUNEL assay demonstrate that 16-month-old mice exhibited a higher abundance of apoptotic cells in comparison to young mice. The result is consistent with the previous studies in aging mice [34, 35]. However, the administration of D+Q treatment effectively suppressed this apoptotic activity in the old mice (Fig. 1I-J). Taken together, our findings indicate that D+Q exhibits the potential for ameliorating the senescence process in the aged small intestine by enhancing the clearance of apoptotic cells.

## Senolytic on villus/crypt structure and NO production in old small intestine

The jejunum is composed of plicae circulares, which are muscular flaps, as well as villi that play a role in absorbing digested products. The mucosal lining of the jejunum exhibits an optimization for maximum absorption by being adorned with villi that projects into the lumen, thereby increasing the available surface area. The absorption capacity is directly influenced by the dimensions and density of these villi structures [5]. In this study, GSEA results reveal the POSITIVE regulation of multicelluar organism growth and maintenance of gastrointestinal epitheliun in the responses to D+Q treatment in 14-month-old small intestine (Fig. 1F-H). We confirmed these results by measuring the height and number of villi in jejunum in 3-month-old mice and 16-month-old mice treated with/without D+Q. In accordance with previous research [7, 36], we have observed a deterioration in the structural integrity of villi in the jejunum of 16-month-old mice, as evidenced by a significant reduction in both villi height and quantity. Nevertheless, administration of D+Q effectively mitigated the reductions observed in the height and number of villi and crypts in aged mice (Fig. 2A-B and Fig. 2D-E).

Aged mice exhibited a decrease in the height and number of crypts, which house intestinal stem cells (ISCs) and progenitor cells involved in controlling the size and density of villi [6, 37]. Our results indicate that treatment with D+Q was effective in reversing the reductions in both villi and crypts (Fig. 2A-F). Additionally, as progenitor cells transition into absorptive or secretory cells and migrate towards the base of the villi, cell proliferation ceases as differentiated cells leave the crypt [38, 39]. We observed a decrease in the number of Ki67+ progenitor cells in old mice compared to young mice, which were rescued by D+Q treatment (Fig. 2G-H).

Nitric oxide exerts multiple effects on the functionality of intestinal epithelial cells through intricate intracellular and molecular mechanisms of action. Achieving a balanced redox status is of pivotal significance for the preservation of pathways crucial for the functionality of intestinal epithelial cells [40]. Modulating nitric oxide in order to sustain cellular redox homeostasis represents an essential underlying mechanism for ensuring optimal function of intestinal tight junctions [40]. Hence, an examination of nitric oxide levels was conducted in the small intestine following treatment with either a vehicle or D+Q. Consistent with previous studies that nitric oxide production impaired in intestine during aging [41], interestingly, our findings demonstrated a significantly augmented nitric oxide concentration in the small intestine treated with D+Q, as compared to the cells treated with the vehicle group (Fig. 2I-J).

In summary, our investigation provides evidence supporting the potential of implementing senolytic cocktail D+Q as a means to revive senescent small intestines. This rationale is likely attributed to the ability of D+Q to mitigate the occurrence of programmed cell death in the epithelial cells of the small intestine, facilitate the multiplication of progenitor cells within crypts, consequently leading to a mitigation of the deterioration observed in the structural integrity of the small intestine's epithelium. These findings provide the possibility for clinical reversal of intestinal aging in elderly through drugs. With the inclusion of other senolytic compounds, these agents exhibit promising prospects in enhancing the functionality of the small intestine in elderly populations, potentially improving nutritional status, healthspan, and remaining survival in aged subjects, but this speculation requires substantial further testing. Future *in vivo* and *in vitro* experiments will be carried out to investigate the molecular mechanism of D+Q in promoting the proliferation of progenitor cells in the small intestinal epithelial crypts to provide a theoretical basis for its clinical application against intestinal aging.

## Data Availability Statement

The datasets used and/or analyzed during the current study are available from the corresponding author upon reasonable request.
